# Flexible Asymmetric Organic-Inorganic Composite Solid-State Electrolyte Based on PI Membrane for Ambient Temperature Solid-State Lithium Metal Batteries

**DOI:** 10.3389/fchem.2022.855800

**Published:** 2022-03-23

**Authors:** Ruilu Yang, Zheng Zhang, Qi Zhang, Jian Shi, Shusen Kang, Yanchen Fan

**Affiliations:** ^1^ Analysis and Testing Center, Nantong University, Nantong, China; ^2^ SUSTeach Academy for Advanced Interdisciplinary Studies and Department of Materials Science and Engineering, Southern University of Science and Technology, Shenzhen, China

**Keywords:** Asymmetrical structure, solid-state electrolyte, solid-state lithium metal batteries, Poly(ethylene glycol), PI membrane

## Abstract

Solid-state lithium metal batteries have attracted more and more attention in recent years because of their high safety and energy density, with developments in the new energy industry and energy storage industry. However, solid-state electrolytes are usually symmetric and are not compatible with the cathode and anode at once. In this work, a flexible asymmetric organic-inorganic composite solid-state electrolyte consisting of PI membrane, succinonitrile (SN), LiLaZrTaO(LLZTO), Poly (ethylene glycol) (PEO), and LiTFSI were prepared by solution casting successfully. This lightweight solid electrolyte is stable at a high temperature of 150°C and exhibits a wide electrochemical window of more than 6 V. Furthermore, the high ionic conductivity of the flexible solid electrolyte was 7.3 × 10^−7^ S/cm. The solid-state batteries assembled with this flexible asymmetric organic-inorganic composite solid electrolyte exhibit excellent performance at ambient temperature. The specific discharge capacity of coin cells using asymmetric organic-inorganic composite solid-state electrolytes was 156.56 mAh/g, 147.25 mAh/g, and 66.55 mAh/g at 0.1, 0.2, and 1C at room temperature. After 100 cycles at 0.2C, the reversible discharging capacity was 96.01 mAh/g, and Coulombic efficiency was 98%. Considering the good performance mentioned above, our designed flexible asymmetric organic-inorganic composite solid electrolyte is appropriate for next-generation solid-state batteries with high cycling stability.

## Introduction

Lithium-ion batteries (LIB) are widely applied in electric vehicles, portable devices, and smart grids because of their good performance (Lu et al., 2013; Humana et al., 2016; Liu et al., 2019; Sheng et al., 2019). In recent decades, researchers around the world have made tremendous progress on every component of batteries. However, the safety problems of high energy density LIB remain a problem (Wang et al., 2012; Eshetu et al., 2013; Xie and Lu, 2020), especially because the lithium metal anode is expected to be used in the high energy LIB, as more serious safety issues affect its highest capacity density and lowest potential. Li dendrites formed during electrochemical Li plating and stripping can penetrate the separator, leading to battery short-circuiting, and eventually fire and tragedies (Zhang, 2018; Wan et al., 2019; Wang et al., 2019).

To improve the safety of LIB, solid-state batteries have attracted significant attention, because they can replace the flammable liquid electrolyte in the current LIB (Jung et al., 2016; Gai et al., 2019; Xiao et al., 2020; Yu et al., 2020; Yu et al., 2021). The key component of solid-state batteries is a solid-state electrolyte (SSE). A perfect SSE should have high ionic conductivity, good interfacial stability and adhesion with the electrodes, a wide electrochemical window, good chemical stability, strong mechanical stability, nonvolatility, and nonflammability (Quartarone and Mustarelli, 2011; Judez et al., 2019; Xia et al., 2022). A great deal of research has been devoted to the various SSE materials, which can be summarized into three kinds, inorganic (oxides/sulfide) solid electrolytes, solid polymer electrolytes (SPEs), and their hybrids. There are advantages and disadvantages to the three kinds of solid-state electrolytes (Porz et al., 2017; Chua et al., 2018; Sen et al., 2021).

The ionic conductivity of inorganic SSEs is the highest. Some sulfide SSEs even have higher ionic conductivity than that of liquid electrolytes (Lian et al., 2019; Reddy et al., 2020). While there is large interfacial resistance in solid-state lithium metal batteries, which has prompted the leap in SSEs towards applications in lithium metal batteries. The inorganic SSEs are not flexible and thick. An increasing numbers of studies have found that the intrinsic high electronic conductivity in certain inorganic SSEs, especially at grain boundaries, leads to hazardous direct Li deposition inside of them (Dawson et al., 2017; Soares et al., 2019). Polymer electrolytes are flexible, lightweight, and have easy scalability (Manthiram et al., 2017). SPEs contain polymers, Li salts, and plasticizers. However, their application was limited by moderate ionic conductivity and narrow electrolyte windows. The low mechanical strength of the SPEs hinders their practical application in electric vehicles. Polyethylene oxide solid-state electrolyte system is the most widely studied, while the PEO itself is flammable. This is a giant leap to its practical application in LIBs (Zhang et al., 2016; Ding et al., 2021). A composite solid electrolyte containing organic and inorganic solid electrolytes has attracted more and more attention because of its high ionic conductivity and flexibility. However, the ionic conductivity of the composite solid electrolyte is usually below 10^−4^ S/cm and cannot be applied in solid-state batteries at room temperature.

It is therefore necessary to develop a solid-state electrolyte with higher conductivity. A solid-state electrolyte based on the succinonitrile (SN) exhibited outstanding ion transport in a quasi-solid state because its melting point is 58°C (Chen et al., 2016). Solid-state electrolytes containing lithium salt and SN exhibit ionic conductivity over 10^−3^ S/cm, which is much higher than these of SPEs. Moreover, the SN is electrochemically stable and negligible flammable, which is suitable for lithium batteries. However, the mechanical property of SN is poor. For solid-state LIBs, available SSEs need high mechanical strength, wide electrochemical windows, and high ionic conductivity to satisfy the solid-state LIBs.

However, the SSEs are symmetric because of the preparation method. Inorganic SSEs usually are prepared by solid-state sintered technology. The polymer SSEs are often prepared by solution-casting. The symmetric SSEs are not compatible with the cathode and anode at once. The cathode is oxidative, and the anode is reductive. The PEO-based SSEs are compatible with lithium metal anode, but they can be oxidized by a high-voltage cathode. LATP has high ionic conductivity, and the titanium in LATP can be reduced from +4 to +3 when in direct contact with metallic lithium, meaning the symmetric SSEs cannot safely be used in solid-state lithium metal batteries.

The present study proposes a design for a flexible asymmetric organic-inorganic composite solid-state electrolyte for all solid-state LIBs. The composite SSE contains polyimide film, polymer, ceramic electrolyte, SN, and a lithium salt. Polyimide film has an aligned porous and high mechanical strength to guarantee the structural integrity and good flexibility of the electrolyte to prevent potential dendrite penetration. The aligned composite SSEs structures in a horizontal configuration could enhance the ionic conductivity. The polymer in the SSEs could reduce dendrite growth. PEO solid-state electrolytes are the highest ionic conductivity and are applied at the organic-inorganic composite solid-state electrolyte. Ceramic electrolyte benefits for the high electrolyte window and ionic conductivity. LLZTO are significant for ceramic electrolyte power because of their excellent stability, high ionic conductivity (higher than 10^−3^ S/cm), and mechanical strength. LLZTO was thus used in the organic-inorganic composite solid-state electrolyte. SN was chosen as the plasticizer because of its high ionic conductivity, electrochemically stability, and nonflammability. Moreover, the SN could acquire a good interfacial contact between SSEs and Li metal anode. The solid-state electrolyte solution can permeate the PI membrane to form the organic-inorganic composite solid-state electrolyte. Utilizing the organic-inorganic composite solid-state electrolyte, the solid-state Lithium batteries can stably cycle at room temperature, which is dramatically high. All in all, the design idea of the composite electrolyte in this work could provide some guidance and opportunities for the novel design of high-performance solid-state lithium metal batteries.

## Experimental Section

### Materials and Chemical Reagents

Poly (ethylene glycol) (PEO, Mw = 8,000) was bought from Aladdin. Actone (99.5%) was purchased by Merck. Succinonitrile (SN, 99.7%), LiTFSI (99.9%) were bought from Energy Chemical. PI membrane was bought from Aladdin. Li_6.4_La_3_Zr_1.4_Ta_0.6_O_12_ (LLZTO, dia12*0.8 mm) was purchased from the Kejing company. All the materials were used directly without any treatment.

### Preparation of the Composite Separator

Firstly, 400 mg PEO, 300 mg SN, and 300 mg LLZTO particles were added to a beaker. Then, some acetone was added into this beaker and stirred at room temperature for 12 h to get a homogenous solution. Next, the homogenous solution was coated on Teflon mold with a PI membrane on the bottom. Finally, this membrane was dried 24 h at room temperature to evaporate the solvent and create the flexible asymmetric organic-inorganic composite solid-state electrolyte.

### Preparation of the LiNi_0.8_Co_0.1_Mn_0.1_O_2_ Cathode Electrode

LiNi_0.8_Co_0.1_Mn_0.1_O_2_, acetylene black, PVDF with a weight ratio of 8:1:1 was added in N-methylpyrrolidone (NMP) and stirred for 12 h to get homogenous slurry. Next, the obtained slurry was coated on aluminum current collectors by a doctor blade. Then the electrode was dried at 80°C for 12 h. The cathode electrode was dried at 100°C for 48 h in a vacuum. The active mass loading on the cathode foil was about 5.1 mg cm^−2^.

### Electrochemical Measurements

CHI 660E was used to test the ionic conductivity of the PVDF-HFP/LLZTO composite GPEs. The symmetric cell stainless steel (SS)/GPE/SS was used. The ionic conductivity of GPE was measured by A.C. impedance spectrum (EIS) at room temperature to 100°C with a frequency range from 1 Hz to 1M Hz. The ionic conductivity was calculated by Eq. 1:

$$б=d/\left(S\times R_{b}\right)$$ (1)

б represents the ionic conductivity, d is the thickness of the GPE, S is the area of polymer electrolyte. AC impedance and DC polarization methods were combined to measure the lithium transference number. The symmetric cell system Li/GPE/Li was used in this test. The lithium transference number was calculated by the following Equation 2

$$t_{Li^{+}}=\frac{I_{s}\left(\Delta V-I_{0}R_{0}\right)}{I_{0}\left(\Delta V-I_{0}R_{0}\right)}$$ (2)

I_0_ represents the initial polarization currents; Is represents the steady-state polarization currents; R_0_ represents the initial interfacial resistance; R_0_ represents the steady-state interfacial resistance; and the voltage amplitude is set at 0.1 V.

SS/GPE/Li was used to tests the electrochemical window by linear sweep voltammetry. Voltage ranges from 0 to 5 V with a rate 0.1 mV/s. The compatibility of the GPE with Li metal was analyzed by plating/stripping cycling experiment of Li metal electrode in Li/GPE/Li cell. The cyclic voltammetry was performed by CHI 660E and ranged from 2.5 to 4.5 V with a scanning rate of 0.1 mV/s at room temperature.

The morphologies of the GPE membrane were characterized by scanning electron microscopy (EVO-18). The X-ray diffraction patterns of the polymer membranes were obtained with X-ray power diffraction (X pert3 Power) at a scanning rate of 4°/min in the 2θ range of 5–90°. The porosity of the polymer membrane was measured by gravimetry. The electrochemical properties of GPE were carried out using CHI660E.

## Results and Discussion

**FIGURE 1:**
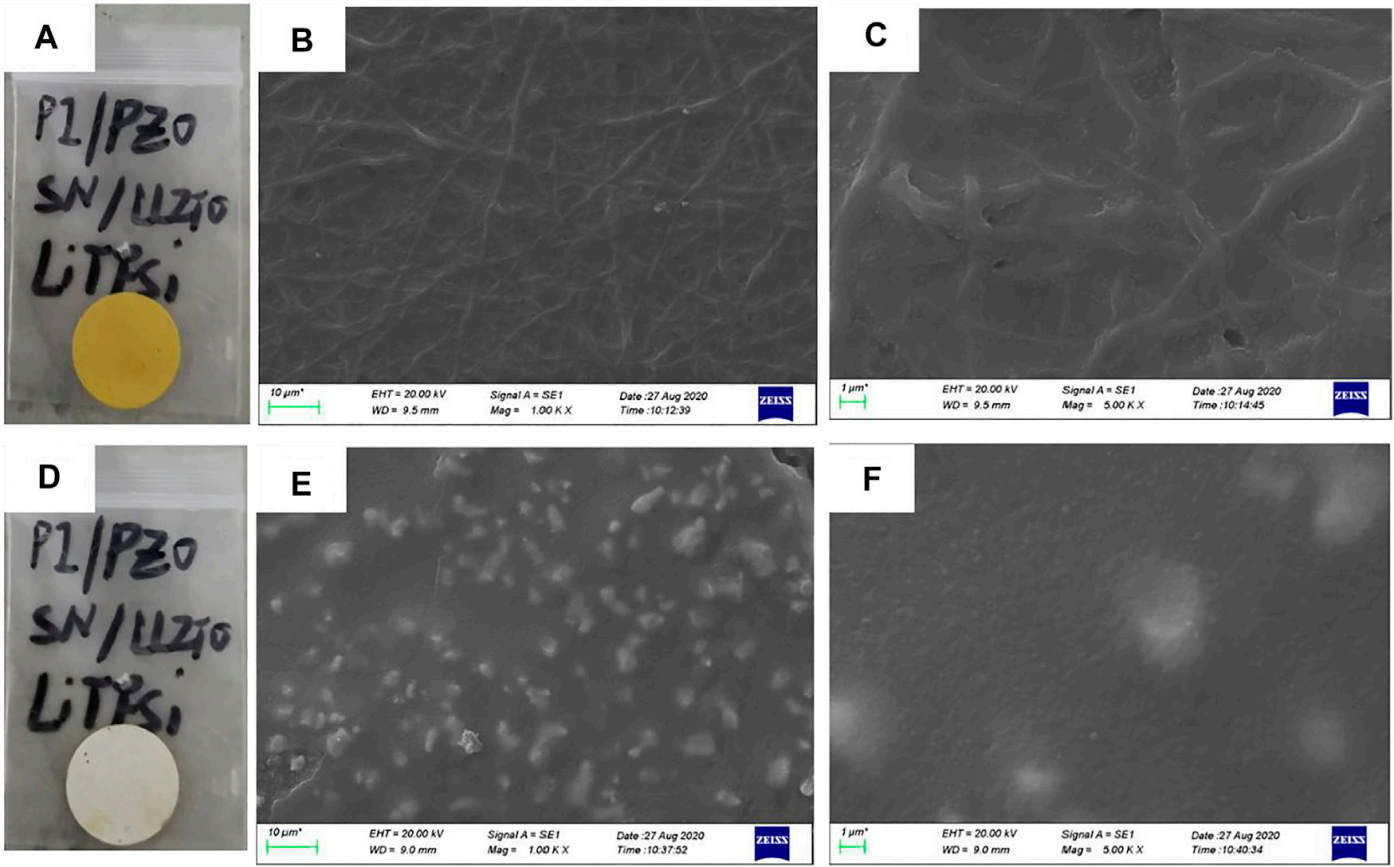
Image and SEM image of asymmetric organic-inorganic composite solid-state electrolyte, **(A), (B), and (C)** are images of the bottom-surface, **(D), (E), and (F)** are the images of the top-surface.

**FIGURE 2:**
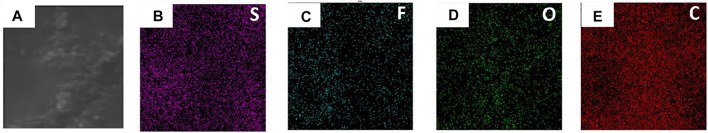
SEM and EDS mapping of the bottom surface of the flexible asymmetric organic-inorganic composite solid-state electrolyte. **(A)** SEM image, **(B)** EDS mapping image of S, **(C)** F, **(D)** O, **(E)** C.

The two sides of the asymmetric organic-inorganic composite solid-state electrolyte have different morphology, which can be seen in Figure 1. The bottom-surface is homogenous and nonporous, and the PI fiber can be seen from the SEM images (Figures 1B,C). The corresponding EDS mapping images show that S, F, O, and C are dispersed evenly in the electrolyte, as indicated in Figure 2. This demonstrates the PEO, SN, and PI are homogeneous on the bottom-surface, and that this side has no LLZTO nanoparticles.

**FIGURE 3:**
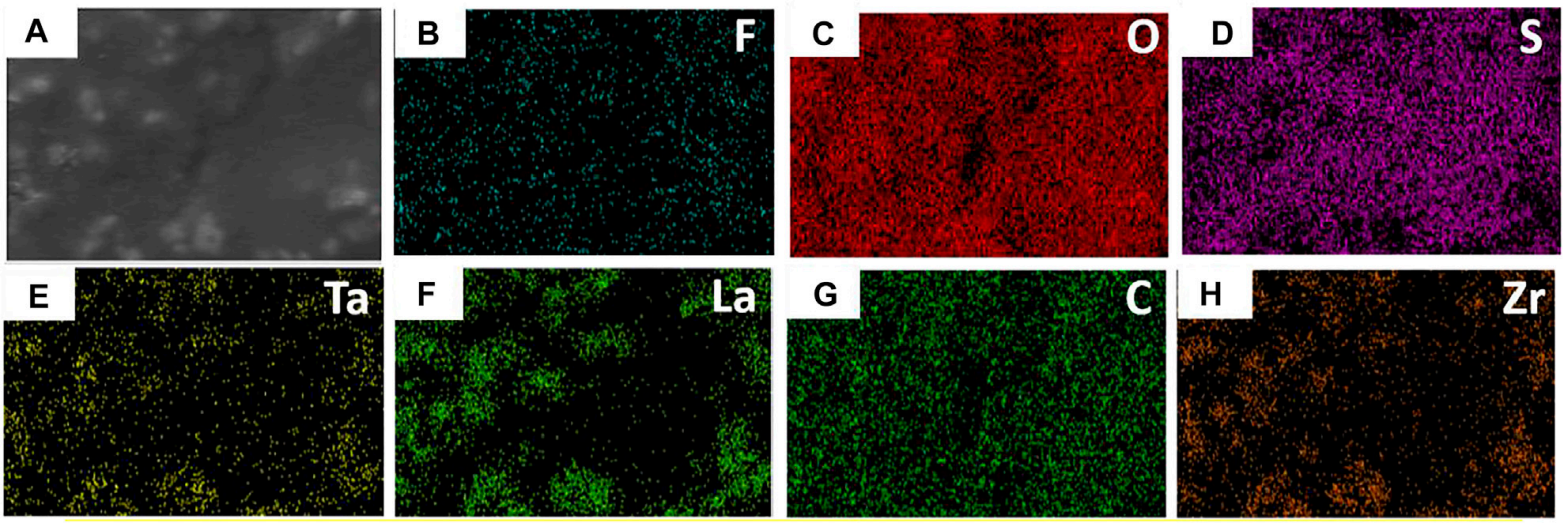
SEM and EDS mapping of the top surface of the flexible asymmetric organic-inorganic composite solid-state electrolyte.**(A)** SEM image, **(B)** EDS mapping image of F, **(C)** O, **(D)** S, **(E)** Ta, **(F)** La, **(G)** C, **(H)** Zr.

**FIGURE 4:**
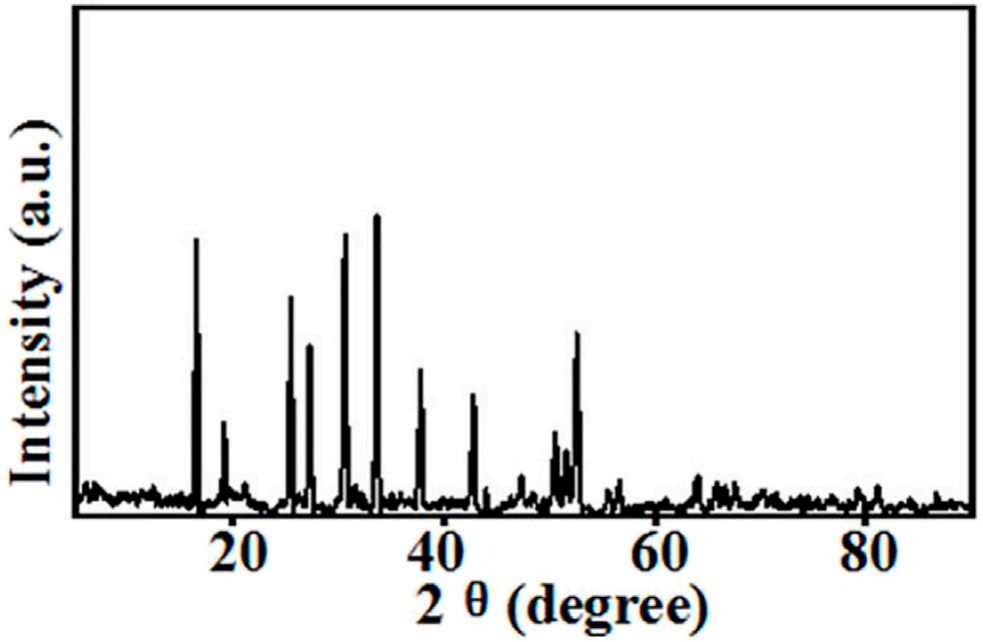
XRD of flexible asymmetric organic-inorganic composite solid-state electrolyte.

**SCHEME 1:**
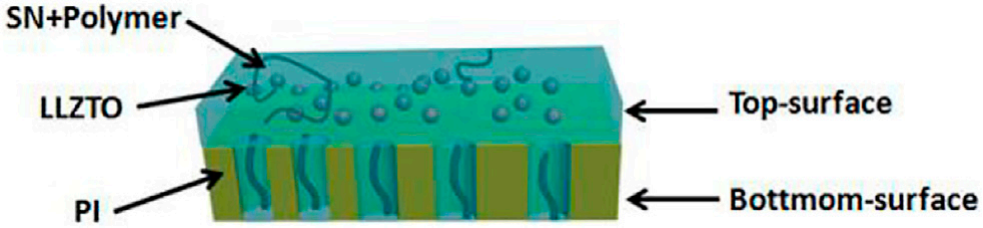
The scheme of asymmetric organic-inorganic composite solid-state electrolyte.

The top-surface is very rough, which can be seen from Figures 1E,F. The original morphology of PI fiber is covered by polymer and nanoparticles, as shown in Figure 3. The corresponding EDS mapping images show that S, F, O, and C are homogenous on the top-surface, and Ti, La, and Zr are evenly distributed on the top-surface because the LLZTOs are nanoparticles. This demonstrates the PEO, SN, LLZTO, and PI are homogeneous on the bottom-surface. The two sides also contain different components. The PI is small, meaning that the LLZTO nanoparticles cannot permeate through to the bottom-surface. The two sides of asymmetric organic-inorganic composite solid-state electrolytes have different morphology, meaning this is a useful way of preparing the two sides of solid-state electrolytes that have different functions. For example, the LATP and LAGP have good ionic conductivity and high electrochemical windows, but they react with lithium metal anode, so they cannot be used in lithium metal batteries. In this way, we can prepare the solid-state electrolyte with two different sides as shown in Scheme 1, one side consists of polymer and lithium salt, so this side is compatible with the lithium metal anode. Another side is made up of nanoparticles, polymer, and lithium salt, so this side is compatible with the high-voltage cathode. Therefore, a solid-state electrolyte prepared in this way is compatible with the high-voltage cathode and a lithium anode. The XRD of the top-surface side is shown in Figure 4. Two board peaks at 18.9 and 20.1 are attributed to the crystal peaks of PEO (Zha et al., 2018). The peaks of garnet LLZTO can be seen from Figure 4 and correspond to Li_5_La_5_Nb_2_O_12_, suggesting that cubic phase LLZTO exists on this side (Wei et al., 2020).

**FIGURE 5:**
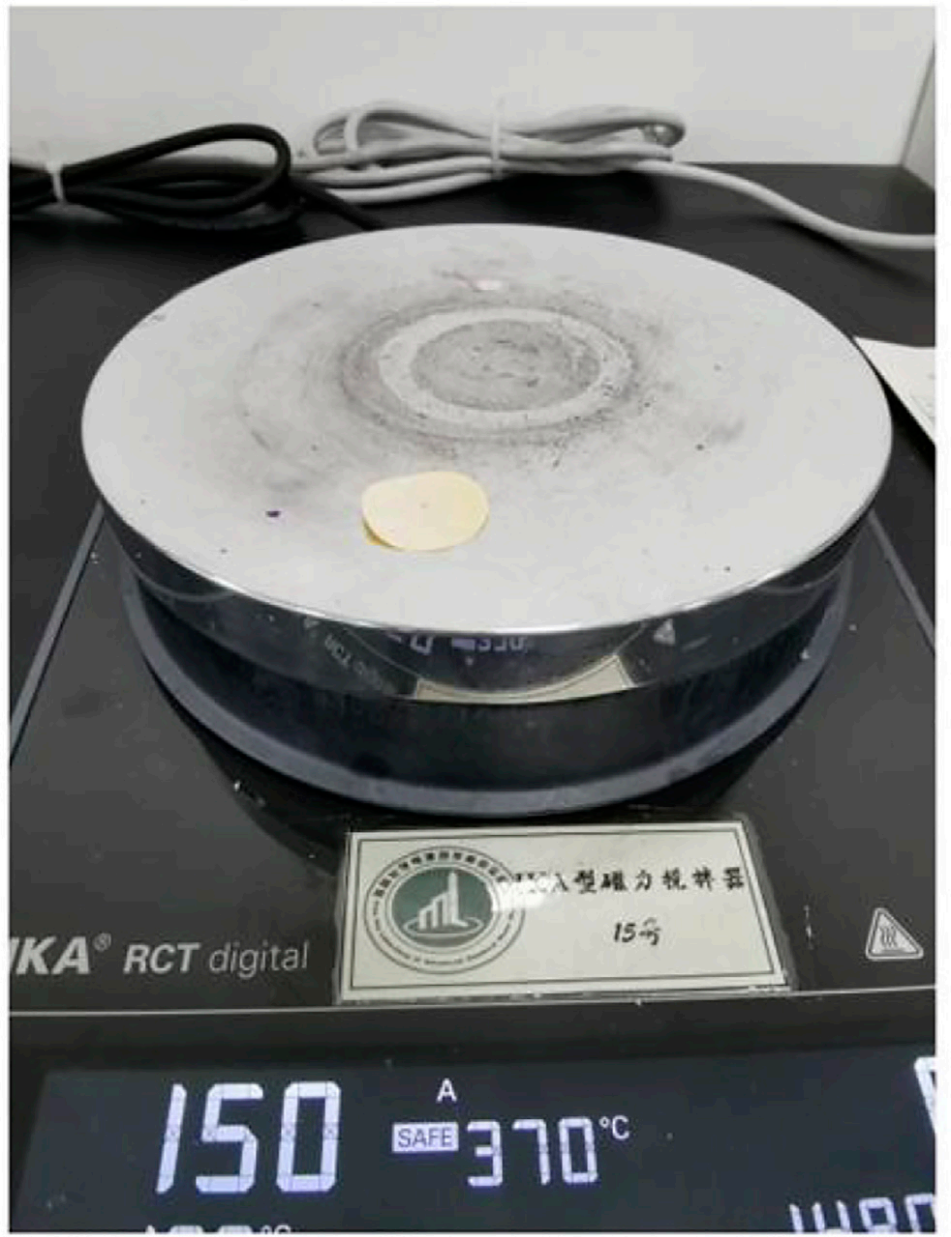
Flexible asymmetric organic-inorganic composite solid-state electrolyte is heated at 150°C for 10 min.

As the safety problems of batteries attract more and more attention, the thermal stability of the asymmetric organic-inorganic composite solid-state electrolyte was evaluated. As shown in Figure 5, the asymmetric organic-inorganic composite solid-state electrolyte showed excellent thermal stability at 150°C. The electrolyte membrane shows no significant change in size and color before and after treatment at 150°C. This is attributed to the high thermal stability of LLZTO (Lu et al., 2019) and PI (Munakata et al., 2008).

**FIGURE 6:**
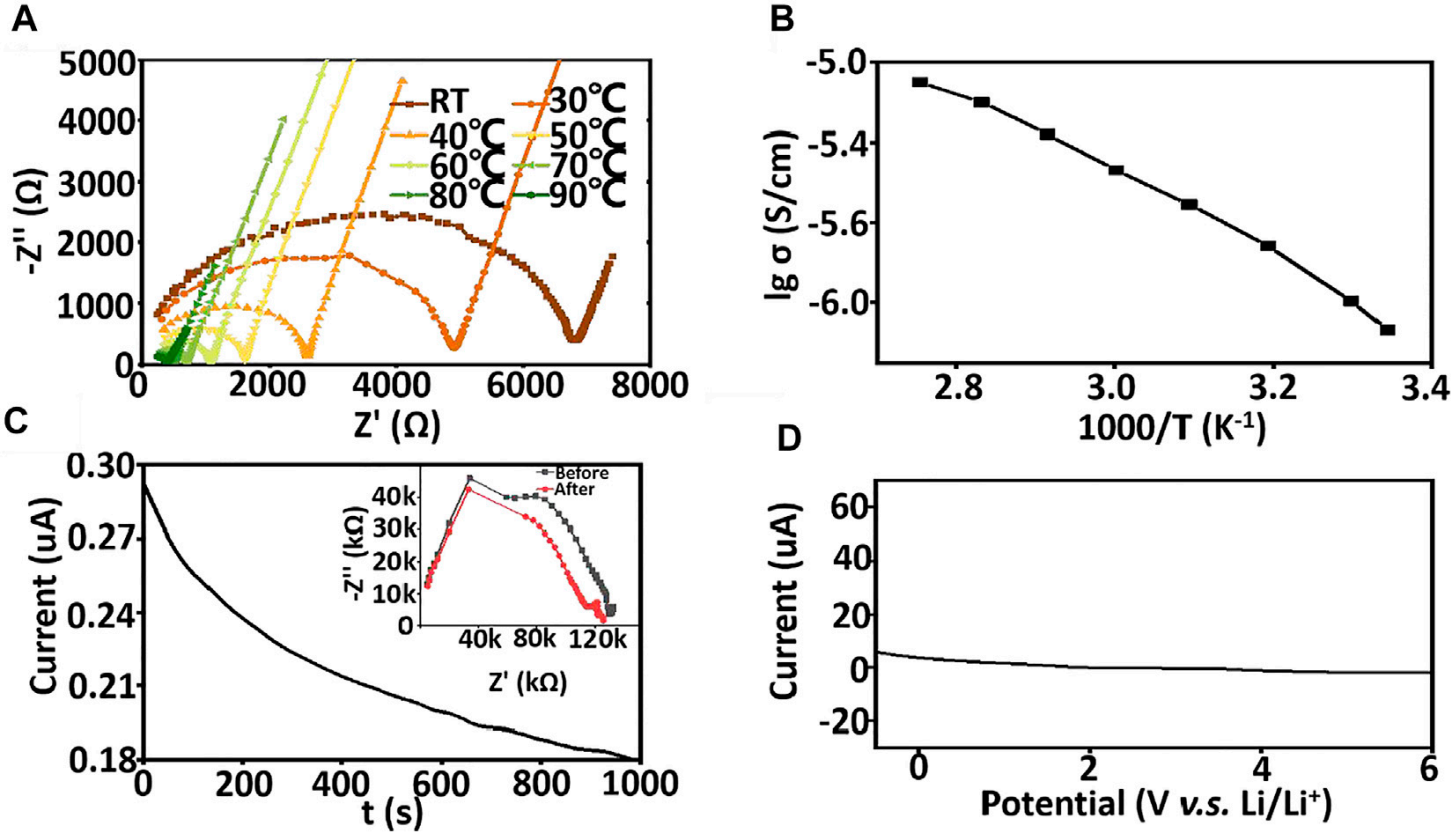
**(A)** the A.C. impendence of asymmetric organic-inorganic composite solid-state electrolyte at different temperature **(B)** Arrhenius plots of asymmetric organic-inorganic composite solid-state electrolyte ranging room temperature to 90°C, **(C)** DC polarization curve for Li/ asymmetric organic-inorganic composite solid-state electrolyte/Li under a polarization voltage of 0.1 V. The insets show the EIS before and after the polarization. **(D)** LSV curve of asymmetric organic-inorganic composite solid-state electrolyte.

The electrochemical properties of the asymmetric organic-inorganic composite solid-state electrolyte are investigated in Figure 6. The ionic conductivity of the asymmetric organic-inorganic composite solid-state electrolyte is 7.3 × 10^−7^ S/cm, which is higher than PEO/LLZTO/LiTFSI solid-state electrolyte (Figure 6A). The polymers are aligned in the PI holes, the ionic diffusion along the aligned direction shows an even bigger difference to those in the other two directions (x and y) in the aligned PEO/LiTFSI system (Wan et al., 2019). Therefore, polymer-chain alignment is beneficial to ion diffusion in the alignment direction and the ionic conductivity of asymmetric organic-inorganic composite solid-state electrolyte increases with the increasing temperature. In Figure 6B, the activation energy was also calculated by Arrhenius theory and was 39.4 kJ/mol. The lithium transference number is characterized by AC impedance and the DC polarization method Figure 6C. The lithium transference number is 0.40. The LSV is used to characterize the electrochemical window. As shown in Figure 6D, there is no oxide peak below 6 V, which is higher than the usual solid-state electrolyte because the SN and LLZTO are electrochemically stable. Therefore, the asymmetric organic-inorganic composite solid-state electrolyte is a satisfactory candidate for high-voltage lithium batteries.

**FIGURE 7:**
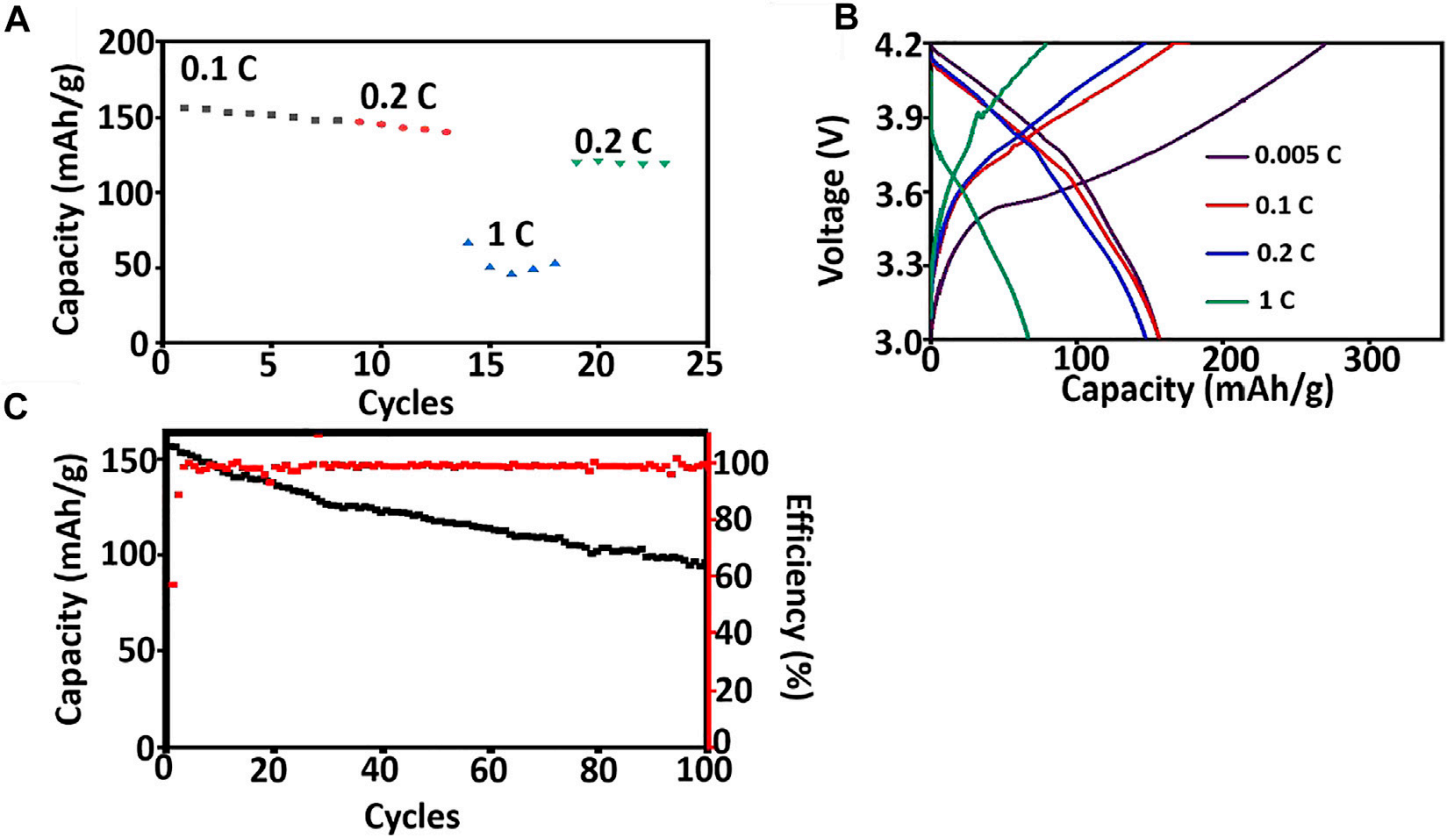
Battery performance of NCM811/ asymmetric organic-inorganic composite solid-state electrolyte/Li at room temperature. **(A)** discharging capacity at different rates, **(B)** charging and discharging curves at different rates, **(C)** long term cycling performance at 0.2 C.

Asymmetric organic-inorganic composite solid-state electrolytes are applied at NCM811/Li to evaluate the possible application in lithium metal batteries. The coin cell is charging and discharging at room temperature to monitor its rate and cycle performance and the results are shown in Figure 7. The specific discharge capacity of coin cells using Asymmetric organic-inorganic composite solid-state electrolytes is 156.56 mAh/g, 147.25 mAh/g, and 66.55 mAh/g at 0.1, 0.2, and 1C at room temperature. This is better than the solid-state lithium metal batteries using PEO/LLZTO composite solid-state electrolyte (Zhang et al., 2015; Zhao et al., 2017). After the discharging rate is changed to 0.2C, the specific discharge capacity is 120.45 mAh/g. Furthermore, the coin cell also exhibits good cycle performance. After 100 cycles, the reversible discharging capacity is 96.01 mAh/g, and Coulombic efficiency is 98%. The excellent cycle performance is attributed to the good interface performance between electrolyte and Li metal anode (Wang et al., 2017).

**FIGURE 8:**
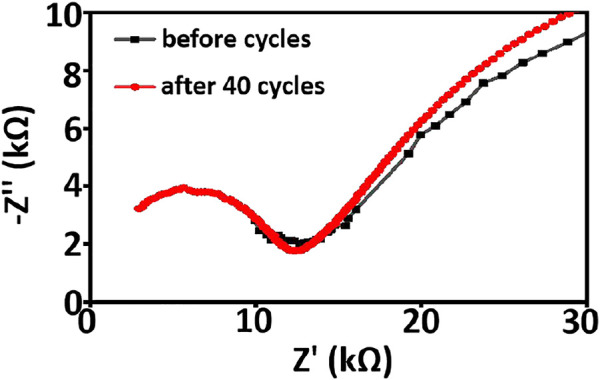
EIS spectroscopy of NCM811/ asymmetric organic-inorganic composite solid-state electrolyte/Li cell before and after 40 cycles.

**FIGURE 9:**
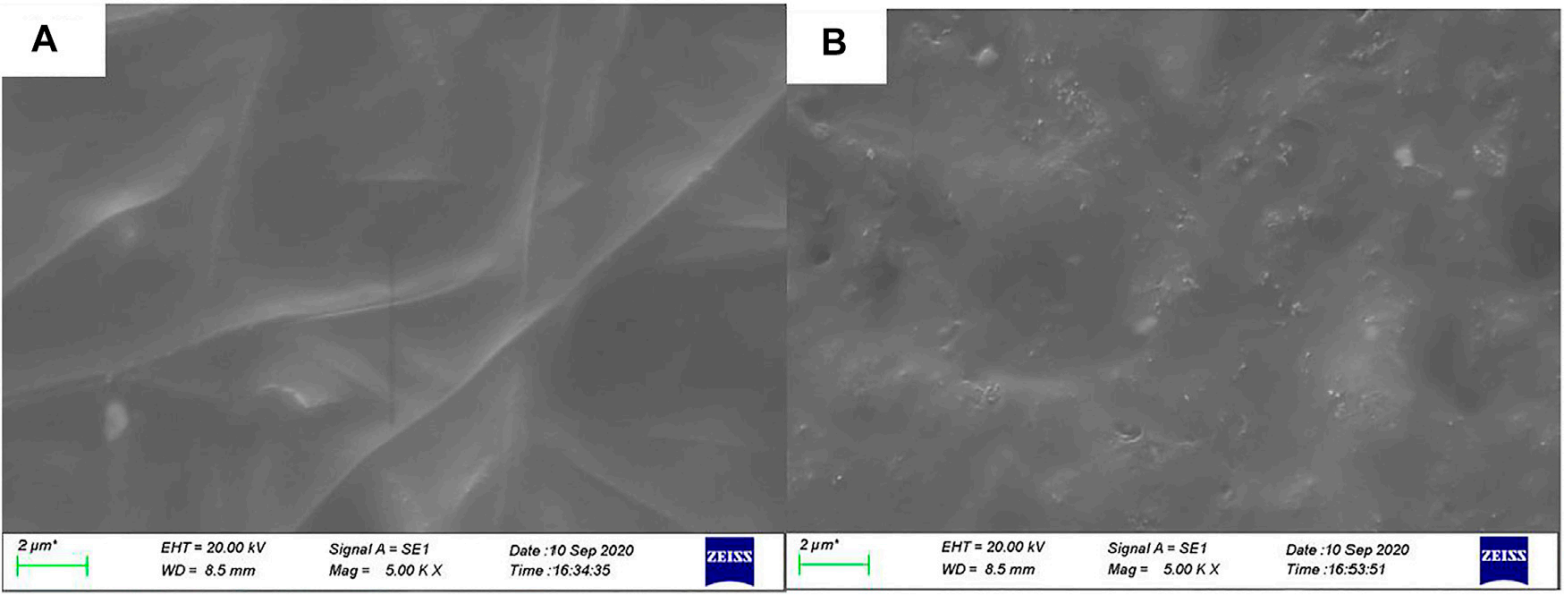
**(A)** bottom-surface **(B)** top-surface SEM of the asymmetric organic-inorganic composite solid-state electrolyte after 40 cycles.

Electrochemical impedance spectroscopy and cyclic voltammetry are used to evaluate the stability of cells with asymmetric organic-inorganic composite solid-state electrolytes. In the EIS spectrum (Figure 8), we can see the interfacial resistance and charge transfer resistance of the cell do not change obviously before and after 40 cycles. This demonstrates that the electrolyte/electrode interface and passivation layer on the electrode is stable and the asymmetric organic-inorganic composite solid-state electrolyte is compatible with the cathode and anode at once. This result corresponds to the result in Figure 9. Illustrated in Figure 9 are the SEM images of the bottom-surface and top-surface of the asymmetric organic-inorganic composite solid-state electrolyte after 40 cycles. Compared with the corresponding SEM images in Figures 1B,C, the bottom-surface is still homogenous and nonporous after 40 cycles. Moreover, the original morphology of PI fiber is still covered by polymer and nanoparticles on the top-surface of the asymmetric organic-inorganic composite solid-state electrolyte after 40 cycles. These findings confirm that the electrolyte/electrode interface and passivation layer on the electrode is stable and the two sides of the asymmetric organic-inorganic composite solid-state electrolyte contain different components.

According to the above analysis, the two sides of asymmetric organic-inorganic composite solid-state electrolytes have different morphology and functions. This is very useful in solid-state electrolytes. We can prepare the solid-state electrolyte with two different sides, one side consists of polymer and lithium salt, so this side is compatible with the lithium metal anode. The other side is made up of nanoparticles, polymer, and lithium salt, so this side is compatible with the high-voltage cathode. Therefore, the solid-state electrolyte prepared in this way is compatible with the high-voltage cathode and a lithium anode. Our designed flexible asymmetric organic-inorganic composite solid electrolyte undoubtedly provides more convenience for the next-generation solid-state batteries with high cycling stability. Furthermore, the design idea of the composite electrolyte in this work can provide some guidance and opportunities for the novel design of high-performance solid-state lithium metal batteries.

## Conclusion

The flexible asymmetric organic-inorganic composite solid-state electrolyte consisting of PI membrane, succinonitrile (SN), LiLaZrTaO(LLZTO), PEO, and LiTFSI were prepared successfully. The flexible asymmetric composite solid-state electrolyte is compatible with the cathode and anode at once. The solid-state batteries assembled with this flexible asymmetric organic-inorganic composite solid electrolyte exhibit excellent performance at ambient temperature. The specific discharge capacity of coin cells using asymmetric organic-inorganic composite solid-state electrolytes is 156.56 mAh/g, 147.25 mAh/g, and 66.55 mAh/g at 0.1, 0.2, and 1 C at room temperature. After 100 cycles at 0.2 C, the reversible discharging capacity is 96.01 mAh/g, and Coulombic efficiency is 98%. Considering the good performance mentioned above, our designed flexible asymmetric organic-inorganic composite solid electrolyte is appropriate for the next-generation solid-state batteries with high cycling stability.

## Data Availability

The original contributions presented in the study are included in the article/supplementary material, further inquiries can be directed to the corresponding authors.
